# Association of teriparatide adherence and persistence with clinical and economic outcomes in Medicare Part D recipients: a retrospective cohort study

**DOI:** 10.1186/1471-2474-14-4

**Published:** 2013-01-03

**Authors:** Leslie Hazel-Fernandez, Anthony M Louder, Shonda A Foster, Claudia L Uribe, Russel T Burge

**Affiliations:** 1 Competitive Health Analytics Inc., Humana Inc, 3501 SW 160 Ave, Miramar, FL, 33029, USA; 2 Eli Lilly and Company, Indianapolis, IN, USA

**Keywords:** Osteoporosis, Teriparatide, Adherence, Persistence, Discontinuation, Medicare part D, Fractures, Outcomes

## Abstract

**Background:**

Improper medication adherence is associated with increased morbidity, healthcare costs, and fracture risk among patients with osteoporosis. The objective of this study was to evaluate the healthcare utilization patterns of Medicare Part D beneficiaries newly initiating teriparatide, and to assess the association of medication adherence and persistence with bone fracture.

**Methods:**

This retrospective cohort study assessed medical and pharmacy claims of 761 Medicare members initiating teriparatide in 2008 and 2009. Baseline characteristics, healthcare use, and healthcare costs 12 and 24 months after teriparatide initiation, were summarized. Adherence, measured by Proportion of Days Covered (PDC), was categorized as high (PDC ≥ 80%), moderate (50% ≥ PDC < 80%), and low (PDC < 50%). Non-persistence was measured as refill gaps in subsequent claims longer than 60 days plus the days of supply from the previous claim. Multivariate logistic regression evaluated the association of adherence and persistence with fracture rates at 12 months.

**Results:**

Within 12 months of teriparatide initiation, 21% of the cohort was highly-adherent. Low-adherent or non-persistent patients visited the ER more frequently than did their highly-adherent or persistent counterparts (*χ*2 = 5.01, p < 0.05 and *χ*2 = 5.84, p < 0.05), and had significantly lower mean pharmacy costs ($4,361 versus $13,472 and $4,757 versus $13,187, p < 0.0001). Furthermore, non-persistent patients had significantly lower total healthcare costs. The healthcare costs of highly-adherent patients were largely pharmacy-related. Similar patterns were observed in the 222 patients who had fractures at 12 months, among whom 89% of fracture-related costs were pharmacy-related. The regression models demonstrated no significant association of adherence or persistence with 12-month fractures. Six months before initiating teriparatide, 50.7% of the cohort had experienced at least 1 fracture episode. At 12 months, these patients were nearly 3 times more likely to have a fracture (OR = 2.9, 95% C.I. 2.1-4.1 p < 0.0001).

**Conclusions:**

Adherence to teriparatide therapy was suboptimal. Increased pharmacy costs seemed to drive greater costs among highly-adherent patients, whereas lower adherence correlated to greater ER utilization but not to greater costs. Having a fracture in the 6 months before teriparatide initiation increased fracture risk at follow-up.

## Background

Osteoporosis is a breakdown and weakening of the bones that affects over 10 million individuals in the U.S. and is linked to increased morbidity, mortality, and healthcare costs [1,2]. Effective medications have been developed to treat osteoporosis, but adherence to treatment is suboptimal [3-5]. Proper adherence may delay and prevent fracture, future bone loss, and other negative health outcomes [6,7]. After one year of therapy, many patients discontinue or decrease dosages of daily oral and injectable medications [7]; this non-persistence and underuse may reduce the drugs’ effectiveness, placing patients at increased risk of fractures and other negative outcomes [1,4,7].

Adherence and persistence with prescribed medication are extremely important for patients at high risk for fractures since these patients are at a much greater risk for fractures relative to patients with mild to moderate osteoporosis. Available studies suggest that reasons for patients’ suboptimal adherence or persistence with osteoporosis medications include concerns about side effects, inconvenience of drug regimens, and drug costs [8,9]. Drug cost concerns are of greater relevance to patients who are prescribed injectable medications because these medications tend to be more costly than oral agents. A recent prospective observational study of patients taking teriparatide, a daily injectable drug developed to treat patients who are at high risk for fractures or for whom first-line treatments have been ineffective [10], found that patients who discontinued therapy within 12 months did so primarily due to problems with paying for prescriptions and perceptions that the benefits of the treatment were outweighed by their concerns about the treatment [11].

The cost sharing attributes of the Medicare Part D prescription drug plan may pose a financial challenge to patients with osteoporosis, especially to those taking costlier medications [12]. Medicare Part D is a part of Medicare, which is a U.S. government-sponsored health insurance that provides coverage primarily for adults over the age of 65. In Medicare Part D, government payments for drug purchases stop when a beneficiary reaches an annual spending limit. Thereafter, beneficiaries must pay for 100% of their prescription costs until their out-of-pocket costs reach a pre-specified threshold (catastrophic coverage) [12,13]. Patients taking costly medications for chronic conditions such as osteoporosis may reach this spending limit sooner, and as a consequence, they may resort to cost-coping behaviors to manage their healthcare spending during the resulting gaps in coverage [14-17]. Some studies of Medicare Part D patients have identified cost-coping strategies such as using medications less frequently than prescribed, discontinuing medications, not filling prescriptions, and switching to less expensive agents [18-20]. Other studies suggest that cost-related responses are more common among patients who have better knowledge of their benefits and who report fewer financial burdens [21,22]. With regard to teriparatide use among Medicare beneficiaries, one study posits that patients’ experiences with the drug affect their healthcare spending habits and their adherence during gaps in coverage [23].

In a recent retrospective cohort study, Tamariz and colleagues evaluated patient persistence with teriparatide and other osteoporosis medications as compared to persistence with biologic therapies used to treat rheumatoid arthritis (RA) and multiple sclerosis (MS) [12]. The authors compared the pharmacy claims of health plan members who had Medicare Part D prescription plans with and without coverage gaps. The findings indicated that patients taking osteoporosis medications, particularly teriparatide, were more likely to discontinue taking their medication if they reached the coverage gap, whereas no such association was observed for patients taking medications to treat RA or MS. A limitation of this study was that it did not assess the medical and economic outcomes associated with these persistence patterns. In another retrospective cohort study, Yu and colleagues evaluated the pharmacy and medical claims of new teriparatide users aged 18 years and older [6]. The authors found that patients’ optimal adherence was associated with a reduced risk of fractures at 6, 12 and 18 months following teriparatide initiation. This study did not specifically assess fracture outcomes among Medicare part D beneficiaries [6].

In order to address gaps noted in previous research, the present study evaluated outcomes associated with adherence and persistence among Medicare Part D beneficiaries. This retrospective investigation was conducted on a cohort of Medicare Advantage with Prescription Drug coverage (MAPD) members who had initiated prescriptions for teriparatide in 2008 and 2009 and who were enrolled through a large national healthcare provider. The study evaluated the outcomes of fracture rates, inpatient/outpatient resource utilization, and direct medical costs.

## Results

### Sample selection

**Figure 1:**
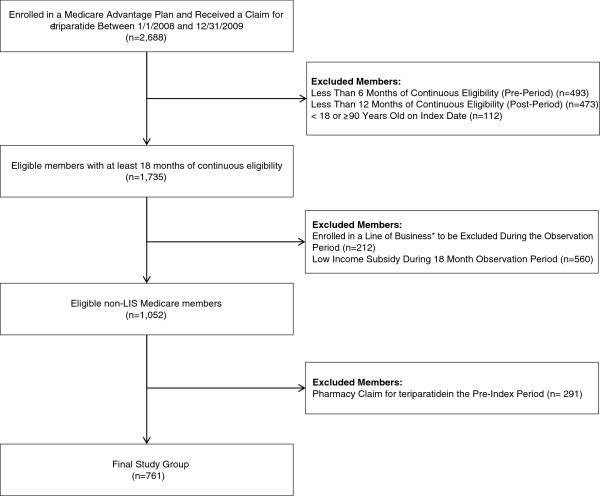
**Sample attrition.** *Line of Business = Type of health insurance coverage.

Figure 1 depicts the attrition diagram which identifies how many health plan members were removed from the sample due to the specified exclusion/inclusion criteria. A total of 2,688 health plan members were enrolled in the Medicare Advantage Plan between January 1, 2008 and December 31, 2009 and were identified as receiving a prescription for teriparatide. Most of those identified were excluded because they were not naïve to teriparatide during the identification period, were not continuously enrolled for at least 6 months pre-index and at least 12 months post index, and/or were eligible for the low income subsidy (LIS) benefit program. The Final Study Group was comprised of 761 members, 36% of which (272 members) had 24 months of continuous post-period eligibility.

### Patient characteristics

Table 1 depicts the baseline demographic characteristics of the final sample (n = 761). The average age of patients was 73 ± 8.4 years, and approximately 66% of the sample was 70 years of age or older. Of the total sample, 88.7% were female, and 93.4% were Caucasian. Participants were primarily from the Southern and Midwestern United States (approximately 66% and 21%, respectively).

**Table 1:** Demographic characteristics

**Characteristic**	**Count**
Total Patients	761
Age, years (Mean, Standard Deviation)	73.3 (±8.4)
Age Distribution (Number, Percent)	
18-29	0
30-39	0
40-49	6 (0.8%)
50-59	31 (4.1%)
60-69	223 (29.3%)
70-79	316 (41.5%)
80-89	185 (24.3%)
Female (Number, Percent)	675 (88.7%)
Ethnicity (Number, Percent)	
White	711 (93.4%)
Black	23 (3.0%)
Hispanic	8 (1.1%)
Other	19 (2.5%)
Geographic Region (Number, Percent)	
Northeast	17 (2.2%)
Midwest	160 (21%)
South	502 (66%)
West	82 (10.8%)

Table 2 summarizes the clinical characteristics of the patient cohort and the percentages of patients who reached the Medicare Part D coverage gap in the 12 months following initiation of teriparatide. At baseline, the Deyo-Charlson score for the group was relatively low (mean of 1.1) indicating a low level of significant comorbidities. The top three most prevalent comorbidities were other disorders of bone and cartilage (ICD-9 code 733.xx; 98.8%), essential hypertension (ICD-9 code 401.xx; 71.7%), and disorders of lipid metabolism (ICD-9 code 272.xx; 69.5%). With regard to the use of osteoporosis drug therapy in the 6 month ‘pre’ period, 46.4% of the cohort (353 patients) had used an osteoporosis medication. Among these users, the most frequently used medications were bisphosphonates (80%), calcitonin (11%), selective estrogen receptor modulator (11%), and conjugated estrogen (6%). With respect to prior fractures, 50.7% of the sample (386 patients) had experienced a fracture during the 6 months before initiation of teriparatide. In the 12-months following initiation of teriparatide, 77% of the sample (586 patients) had experienced a gap in Medicare Part D prescription drug coverage.

**Table 2:** Clinical characteristics

**Characteristic**	**Count**
Total Patients	761
Baseline Clinical Characteristics	
Charlson Comorbidity Index*	1.1 (± 1.6)
	1 [0 - 12]
Osteoporosis Drug Utilization in Pre-Period n,(%)	353 (46.4%)
Bisphosphonate n,(%)	283 (80.2%)
Calcitonin n,(%)	39 (11.0%)
Selective Estrogen Receptor Modulator (SERM) n,(%)	42 (11.9%)
Conjugated Estrogen n,(%)	22 (6.2%)
Fracture in the 6 Month Pre-Period n, (%)	386 (50.7%)
Fracture in the 12 Month Pre-Period n,(%) **	443 (58.2%)
Comorbidity (Top 10 Identified) n, (%)^†^	
Other disorders of bone and cartilage (733.)	752 (98.8%)
Essential hypertension (401.)	546 (71.7%)
Disorders of lipid metabolism (272.)	529 (69.5%)
General symptoms (780.)	474 (62.3%)
Other and unspecified disorders of back (724.)	402 (52.8%)
Symptoms involving respiratory system and other chest symptoms (786.)	391 (51.4%)
Other and unspecified disorders of joint (719.)	382 (50.2%)
Osteoarthritis and allied disorders (715.)	361 (47.44%)
Other disorders of soft tissues (729.)	333 (43.8%)
Cataract (366.)	281 (36.9%)
Post-Period Clinical Characteristics	
Fracture in the 12 Month Post-Period n,(%)	222 (29.2%)
Time Between Index Date and First Fracture (Days)*	200 (± 81)
	181 [91 - 364]
Proportion of Days Covered (PDC) Between Index Date and First Fracture*	0.46 (± 0.3)
	0.38 [0.08 - 1.0]
Reached Part D Coverage Gap During 12 Month Follow-up Period n, (%)	586 (77%)
Out of Pocket Cost for All Prescription Claims During 12 Month Follow-up	$2,082 (± $2,189)
Period*	$991 [$30 - $10,894]
Out of Pocket Cost for Teriparatide Prescription Claims During 12 Month	$1,247 (± $1,832)
Follow-up Period*	$228 [$15 - $7,165]

*Mean (M), standard deviation (SD), median, [range].

** Data for fracture in 12 month pre-period were not available for the entire cohort.

^†^Based on the first 3 digits of ICD-9 CM code.

### Healthcare resource utilization and costs in the follow-up period

Overall resource utilization in the 12 and 24-month period following teriparatide initiation is summarized in Table 3. In the 12-month post period, approximately 21% of the study group had at least 1 hospitalization, 31% had at least one emergency room (ER) visit, 99.9% had at least one outpatient visit, and all members had at least one pharmacy claim. In the subgroup of members with 24 months of post-period follow-up (i.e., members with coverage for months 1-24 following teriparatide initiation; n = 272), 31% had at least 1 hospitalization, more than 44% had ER visits, and all members had at least one outpatient visit and at least one pharmacy claim. As noted in Table 3, the mean total healthcare costs (plan paid and member share combined) amounted to $17,460 per member at 12 months follow-up. At 24 months the costs were $30,292 per member. Inpatient hospitalization costs were on average $4,585 (26%) at 12 months and $8,978 (29%) at 24 months. Outpatient costs were $5,713 (33%) at 12 months and $9,673 (32%) at 24 months. Pharmacy costs allocated to the average member amounted to $6,762 (39%) at 12 months and $10,787 (36%) at 24 months.

**Table 3:** Total healthcare resource utilization and costs

**Measure**	**12 month post period**	**24 month post period**
Total Patients	761	272
Inpatient Hospitalization		
Members with Hospitalization n,(%)	158 (20.8%)	85 (31.3%)
Hospitalizations Per Member**	0.4 (± 0.9)	0.7 (± 1.4)
	0 [0 - 6]	0 [0 - 8]
Emergency Room Visits		
Members with Visit n,(%)	236 (31.01%)	121 (44.49%)
Visits Per Member**	0.6 (± 1.6)	1.3 (± 3.0)
	0 [0 - 30]	0 [0 - 42]
Outpatient Visits		
Members with Visit n,(%)	760 (99.9%)	272 (100.0%)
Visits Per Member**	35. (± 33.5)	68.6 (± 61.7)
	25 [0 - 320]	53 [9 - 698]
Pharmacy Claims		
Members with Pharmacy Claim, n,(%)	761 (100.0%)	272 (100.0%)
Pharmacy Claims Per Member**	45.99 (± 31.9)	98.60 (± 65.9)
	39 [1 - 214]	83 [4 - 366]
		
Total Healthcare Cost**	$17,460 (± $33,437)	$30,292 (± $67,230)
	$10,896 [$1,097 - $758,916]	$18,106 [$2,859 - $1,060,087]
		
Inpatient Hospitalization Cost**	$4,585 (± $23,717)	$8,978 (± $48,891)
	$0 [$0 - $562,430]	$0 [$0 - $774,152]
		
Emergency Room Visit Cost**	$400 (± $2,433)	$855 (± $4,312)
	$0 [$0 - $62,129]	$0 [$0 - $68,565]
		
Outpatient Cost**	$5,713 (± $8,397)	$9,673 (± $12,448)
	$2,724 [$0 - $91,816]	$6,067 [$0 - $144,264]
		
Pharmacy Cost**	$6,762 (± $6,359)	$10,787 (± $10,996)
	$4,253 [$829 - $72,703]	$6,343 [$855 - $73,434]

**Mean, standard deviation, median, [range].

Fracture-related costs (plan cost and member share) are summarized in Table 4 for members identified as having a fracture during the 12- or 24-month post periods (222 and 134 members, respectively). The mean total fracture-related direct costs were $6,198 per member at 12 months (i.e., months 1 to 12), and $8,389 per member at 24 months of follow-up (i.e., months 1 to 24). ER-related costs were, on average, $35 at 12 months and almost triple that amount at $100 at 24 months. In contrast, inpatient hospitalization costs were, on average, $1,123 at 12 months, and were $967 at 24 months. Mean outpatient costs were $829 at 12 months and $1,007 at 24 months. The average osteoporosis medication prescription costs allocated to each member amounted to $3,965 at 12 months and $6,061 at 24 months. Pharmacy costs for acute fracture treatment (pain medication) were close at both time periods at $246 per member at 12 months, and $253 per member at 24 months.

**Table 4:** **Fracture-related healthcare resource utilization and costs**^**†**^

**Measure**	**12 month post period**	**24 month post period**
Total Patients	222	134
Fracture-Related Inpatient Hospitalization		
Members with Hospitalization n,(%)	21 (9.5%)	11 (8.2%)
Hospitalizations Per Member**	0.1 (± 0.4)	0.1 (± 0.4)
	0 [0 - 2]	0 [0 - 2]
Fracture-Related Emergency Room Visits		
Members with Visit n,(%)	35 (15.8%)	33 (24.6%)
Visits Per Member**	0.2 (± 0.5)	0.3 (± 0.6)
	0 [0 - 3]	0 [0 - 3]
Fracture-Related Outpatient Visits		
Members with Visit n,(%)	213 (95.9%)	126 (94%)
Visits Per Member**	4.1 (± 7.4)	4.6 (± 8.55)
	2 [0 - 65]	2 [0 - 61]
Fracture-Related Pharmacy Claims		
Pain Medications		
Members with Pharmacy Claim n,(%)	161 (72.5%)	110 (82.1%)
Pharmacy Claims Per Member**	5.6 (± 7.7)	9.2 (± 11.9)
	2 [0 - 43]	4 [0 - 55]
Osteoporosis Medications		
Members with Pharmacy Claim n,(%)*	222 (100%)	134 (100%)
Pharmacy Claims Per Member**	5.8 (± 4.5)	10. (± 8.7)
	4 [1 - 18]	6 [1 - 29]
		
Total Fracture-Related Cost**	$6,198 (± $6,578)	$8,389 (± $8,650)
	$3,260 [$854 - $40,637]	$3,944 [$854 - $47,033]
		
Inpatient Hospitalization Cost**	$1,123 (± $4,506)	$967 (± $3,582)
	$0 [$0 - $37,355]	$0 [$0 - $22,387]
		
Emergency Room Visit Cost**	$35 (± $117)	$100 (± $674)
	$0 [$0 - $753]	$0 [$0 - $7,692]
		
Outpatient Cost**	$829 (± $2,297)	$1,007 (± $3,128)
	$179 [$0 - $23,383]	$141 [$0 - $23,545]
		
Osteoporosis Medication Cost**	$3,965 (± $3,640)	$6,061 (± $6,737)
	$2,459 [$779 - $14,910]	$2,603 [$784 - $22,965]
		
Pain Medication Cost**	$246 (± $758)	$253 (± $605)
	$24 [$0 - $6,276]	$35 [$0 - $3,575]

^†^ Among patients with a fracture.

*All patients with a fracture had an osteoporosis medication claim.

**Mean, standard deviation, median, [range].

### Description of teriparatide adherence, persistence and economic outcomes

In order to describe the manner in which adherence and persistence with teriparatide therapy were related to economic outcomes, patients were grouped according to their adherence to the drug during the 12-month follow-up period, and whether they persisted with therapy during the same time frame (see Table 5). Adherence status was reported as high (PDC ≥80%), moderate (50% ≤ PDC < 80%), and low (PDC < 50%). Persistence with teriparatide was described as either ‘non-persistent’ or ‘persistent’ with treatment. For each of the defined groups, the overall and fracture-related utilization and costs were calculated. Chi Square analyses, ANOVA pairwise comparisons, and t-tests were used to assess significant differences in healthcare utilization and costs depending on patients’ adherence or persistence groupings.

**Table 5:** Teriparatide utilization and overall utilization and costs in the 12 month follow-up period

**Measure**	PDC* < 50%	**PDC 50% ≤ 80%**	**PDC ≥ 80%**	**Persistent**	**Non-persistent**
	**(low adherent)**	**(moderately adherent)**	**(highly adherent)**		**(60 day gap)**
**Total Patients**	541	57	163	181	580
**Inpatient Hospitalizations**					
**Members with Hospitalization n,(%)**	116 (21.4%)	16 (28.1%)	26 (16.%)	31 (17.13%)	127 (21.9%)
Hospitalizations Per Member**	0.4 (± 0.9)	0.5 (± 1.10)	0.2 (± 0.6)	0.3 (± 0.7)	0.4 (± 0.9)
	0 [0 - 6]	0 [0 - 5]	0 [0 - 4]	0 [0 - 4]	0 [0 - 6]
**Emergency Room Visits**					
**Members with Visit n,(%) Δ ¥**	180 (33.27%)	17 (29.82%)	39 (23.93%)	43 (23.76%)	193 (33.28%)
Visits Per Member**	0.7 (± 1.8)	0.5 (± 0.8)	0.4 (± 1.1)	0.4 (± 1.04)	0.7 (± 1.7)
	0 [0 - 30]	0 [0 - 3]	0 [0 - 9]	0 [0 - 9]	0 [0 - 30]
**Outpatient Visits**					
**Members with Visit n,(%)**	540 (99.8%)	57 (100.0%)	163 (100.0%)	181 (100.0%)	579 (99.8%)
Visits Per Member**	35.4 (± 34.2)	39. (± 39.8)	32.3 (± 28.6)	32.7 (± 29.7)	35.7 (± 34.6)
	26 [0 - 320]	24 [1 - 192]	23 [3 - 195]	23 [3 - 195]	26 [0 - 320]
**Pharmacy Claims**					
**Members with Pharmacy Claim n,(%)**	541 (100.0%)	57 (100.0%)	163 (100.0%)	181 (100.0%)	580 (100.0%)
Pharmacy Claims Per Member** † ‡	44.36 (± 31.01)	43.96 (± 28.70)	52.12 (± 35.06)	51.20 (± 34.72)	44.37 (± 30.79)
	38 [1 - 214]	37 [4 - 127]	41 [6 - 186]	40 [6 - 186]	39 [1 - 214]
					
Total Healthcare Cost** † ‡	$15,528 (± $37,713)	$25,574 (± $28,620)	$21,033 (± $13,136)	$21,046 (± $13,560)	$16,341 (± $37,485)
	$7,272 [$1,097 - $758,916]	$15,124 [$6,403 - $172,809]	$15,777 [$9,745 - $87,302]	$15,777 [$7,901 - $87,302]	$7,562 [$1,097 - $758,916]
					
Inpatient Hospitalization Cost**	$4,897 (± $26,941)	$8,448 (± $21,125)	$2,198 (± $7,342)	$2,535 (± $7,961)	$5,224 (± $26,775)
	$0 [$0 - $562,430]	$0 [$0 - $123,486]	$0 [$0 - $57,410]	$0 [$0 - $57,410]	$0 [$0 - $562,430]
					
Emergency Room Visit Cost**	$465 (± $2,840)	$330 (± $971)	$211 (± $711)	$205 (± $686)	$461 (± $2,759)
	$0 [$0 - $62,129]	$0 [$0 - $5,486]	$0 [$0 - $6,124]	$0 [$0 - $6,124]	$0 [$0 - $62,129]
					
Outpatient Cost**	$5,806 (± $8,860)	$6,439 (± $8,880)	$5,151 (± $6,428)	$5,118 (± $6,290)	$5,899 (± $8,950)
	$2,689 [$0 - $91,816]	$2,724 [$93 - $44,149]	$2,740 [$0 - $34,442]	$2,583 [$0 - $34,442]	$2,771 [$0 - $91,816]
					
Pharmacy Cost** † ‡	$4,361 (± $5,344)	$10,358 (± $5,657)	$13,472 (± $3,787)	$13,187 (± $3,935)	$4,757 (± $5,597)
	$3,445 [$829 - $72,703]	$8,915 [$5,902 - $40,300]	$12,301 [$9,130 - $34,737]	$12,080 [$5,839 - $34,737]	$3,567 [$829 - $72,703]

* Proportion of days covered.

**Mean, standard deviation, median, [range].

† ANOVA pairwise comparison between PDC < 50% and PDC ≥ 80% groups, p < 0.05.

‡ *t*-test between ‘Persistent’ and ‘Non-Persistent’ (60 Day Gap) groups, p < 0.05.

Δ Chi-square comparison between PDC < 50% and PDC ≥ 80% groups, p < 0.05.

¥ Chi-square comparison between ‘Persistent’ and ‘Non-Persistent’ (60 Day Gap) groups, p < 0.05.

### Teriparatide adherence/persistence and fracture-related resource utilization - one year post index

The results in Table 5 show that of the entire cohort, 21% (163 patients) were highly adherent (PDC ≥ 80%) to their teriparatide therapy, and 24% (181 patients) persisted with taking the medication during the 12 month follow-up period. In addition, the results indicate that for the entire sample, there were significant differences in patients’ ER visits, total healthcare costs, and pharmacy costs depending on patients’ adherence or persistence. With regard to healthcare resource utilization, Chi Square test results revealed that there were significant differences in the number of members with ER visits depending on how adherent members were or whether or not they were persistent with therapy. Patients who fell into the low-adherence category (PDC < 50) were more likely to have ER visits than were those who demonstrated high adherence (PDC ≥ 80), (χ^2^ = 5.10, p < 0.05). Similarly, members who were not persistent were more likely to visit the ER during the study period than were the members who were persistent, (χ^2^ = 5.84, p < 0.05).

With regard to total healthcare costs, the results in Table 5 suggest differences in costs depending on the adherence or persistence category that a patient belonged to. The results of t-tests indicated significant differences in total costs when comparisons were made between members who were not persistent (i.e., discontinued therapy) and those that were persistent. Non-persistent members had significantly lower mean healthcare costs (M = $16,341, SD = $37,485) as compared to those who were persistent (M = $21,046 SD = $13,560, t = 2.54, p < 0.05). ANOVA pair-wise comparison revealed that members with PDC <50 had lower total healthcare costs as compared to those with PDC ≥ 80%. The average total costs during the follow up period were lower in members demonstrating low adherence as compared to those with high adherence, although the differences were not significant (M = $15,528, SD = $37,713 versus (M = $21,033, SD = $13,136, F = 3.35, p = 0.068).

Similar to observations of ER visits and total costs, the results suggested significant differences in pharmacy costs depending on adherence or persistence groupings. The ANOVA pair-wise comparison revealed that the average pharmacy costs of members with low adherence (M = $4,361, SD = $5,344) were significantly lower than the costs of members with high adherence (M = $13,472, SD = $3,787, F = 411.35 p < 0.0001). In addition, t-test results indicated significant differences in pharmacy costs when comparisons were made between members who were non-persistent and those that were persistent. Non-persistent members had lower pharmacy costs (M = $4,757, SD = $5,597) than their persistent counterparts (M = $13,187, SD = $3,935, t =22.57, p < 0.0001).

As shown in Table 6, similar adherence/persistence groupings were evaluated for the 222 patients identified as having a fracture during the 12-month follow-up period. Among this group of patients, nearly 18% (39 patients) were identified as being highly-adherent, and 19% (43 patients) were identified as being persistent with teriparatide therapy during the 12 month follow-up period. With regard to fracture-related resource utilization and costs, there were significant differences noted in total costs and osteoporosis medication costs when low adherent patients were compared those who were highly-adherent, and when non-persistent members were compared with those who were persistent. An ANOVA pair-wise comparison revealed that the members with low adherence had, on average, significantly lower total costs (M = $4,419, SD = $6,018), than did members with high adherence (M = $12,670, SD = $4,963 F = 63.254 p < 0.0001). In addition, t-test results showed significantly lower total costs among non-persistent members (M = $4,756, SD = $6,099), as compared to the costs of persistent members (M = $12,199, SD = $4,932 t =7.44, p < 0.0001).

**Table 6:** Teriparatide utilization and fracture-related utilization and costs in the 12 month follow-up period

**Measure**	PDC* < 50%	**PDC 50% ≤80%**	**PDC ≥ 80%**	**Persistent**	**Non-perstistent**
	**low adherent**	**moderately adherent**	**highly adherent**		**(60 day gap)**
**Total Patients, n (%)**	168 (75.7%)	15 (6.8%)	39 (17.6%)	43 (19.4%)	179 (80.6%)
**Fracture-Related Inpatient Hospitalization**					
**Members with Hospitalization, n (%)**	18 (10.7%)	1 (6.7%)	2 (5.1%)	2 (4.7%)	19 (10.6%)
Hospitalizations Per Member**	0.1 (± 0.4)	0.1 (± 0.3)	0.1 (± 0.2)	0. (± 0.2)	0.1 (± 0.4)
	0 [0 - 2]	0 [0 - 1]	0 [0 - 1]	0 [0 - 1]	0 [0 - 2]
**Fracture-Related Emergency Room Visits**					
**Members with Visit, n (%)**	31 (18.5%)	1 (6.7%)	3 (7.7%)	3 (6.98%)	32 (17.9%)
Visits Per Member**	0.2 (± 0.5)	0.1 (± 0.3)	0.1 (± 0.5)	0.1 (± 0.5)	0.2 (± 0.5)
	0 [0 - 2]	0 [0 - 1]	0 [0 - 3]	0 [0 - 3]	0 [0 - 2]
**Fracture-Related Outpatient Visits**					
**Members with Visit, n (%)**	160 (95.2%)	15 (100.%)	38 (97.4%)	42 (97.7%)	171 (95.5%)
Visits Per Member**	4.2 (± 7.96)	4.3 (± 5.14)	3.8 (± 5.48)	3.4 (± 5.21)	4.3 (± 7.83)
	2 [0 - 65]	2 [1 - 17]	2 [0 - 32]	2 [0 - 32]	2 [0 - 65]
**Fracture-Related Pharmacy Claims**					
**Pain Medications**					
** Members with Pharmacy Claim, n (%)**	126 (75.0%)	11 (73.33%)	24 (61.54%)	27 (62.79%)	134 (74.86%)
Pharmacy Claims Per Member**	5.5 (± 7.3)	6.1 (± 10.2)	5.7 (± 8.4)	5.5 (± 8.4)	5.6 (± 7.5)
	2 [0 - 43]	2 [0 - 33]	2 [0 - 42]	2 [0 - 42]	2 [0 - 43]
**Osteoporosis Medications**					
**Members with Pharmacy Claim, n (%)**	168 (100.%)	15 (100.%)	39 (100.%)	43 (100.%)	179 (100.%)
Pharmacy Claims Per Member** † ‡	4. (± 3.3)	9. (± 1.4)	12.5 (± 2.4)	12.2 (± 2.5)	4.3 (± 3.4)
	3 [1 - 16]	9 [7 - 12]	13 [5 - 18]	12 [5 - 18]	3 [1 - 16]
					
Total Healthcare Cost** † ‡	$4,419 (± $6,018)	$9,292 (± $4,396)	$12,670 (± $4,963)	$12,199 (± $4,932)	$4,756 (± $6,099)
	$2,710 [$854 - $40,637]	$8,111 [$5,850 - $23,884]	$11,296 [$9,008 - $36,026]	$11,263 [$7,616 - $36,026]	$2,765 [$854 - $40,637]
					
Inpatient Hospitalization Cost**	$1,325 (± $4,988)	$830 (± $3,214)	$366 (± $2,026)	$332 (± $1,930)	$1,313 (± $4,913)
	$ [$0 - $37,355]	$ [$0 - $12,449]	$ [$0 - $12,585]	$ [$0 - $12,585]	$ [$0 - $37,355]
					
Emergency Room Visit Cost**	$44 (± $132)	$9 (± $36)	$6 (± $24)	$6 (± $23)	$42 (± $129)
	$0 [$0 - $753]	$0 [$0 - $141]	$0 [$0 - $128]	$0 [$0 - $128]	$0 [$0 - $753]
					
Outpatient Cost**	$767 (± $1,903)	$955 (± $1,538)	$1,048 (± $3,716)	$941 (± $3,545)	$802 (± $1,892)
	$159 [$0 - $12,380]	$220 [$0 - $5,733]	$215 [$0 - $23,383]	$186 [$0 - $23,383]	$176 [$0 - $12,380]
					
Osteoporosis Medication Cost** † ‡	$2,068 (± $1,196)	$7,420 (± $1,169)	$10,810 (± $1,250)	$10,516 (± $1,520)	$2,391 (± $1,746)
	$1,713 [$779 - $5,259]	$7,350 [$5,744 - $9,631]	$10,969 [$8,811 - $14,910]	$10,748 [$6,836 - $14,910]	$1,735 [$779 - $9,631]
					
Pain Medication Pharmacy Cost**	$216 (± $646)	$77 (± $172)	$440 (± $1,201)	$404 (± $1,150)	$208 (± $628)
	$28 [$0 - $6,071]	$11 [$0 - $662]	$18 [$0 - $6,276]	$14 [$0 - $6,276]	$27 [$0 - $6,071]

* Proportion of days covered.

**Mean, standard deviation, median, [range].

† ANOVA pairwise comparison between PDC < 50% and PDC ≥ 80% groups, p < 0.05.

‡ *t*-test between ‘Persistent’ and ‘Non-Persistent’ (60 Day Gap) groups, p < 0.05.

With regard to osteoporosis medication costs, the average costs during the follow up period were significantly lower for members demonstrating low adherence (M = $2,068, SD = $1,196), as compared to the higher costs for those with high adherence (M = $10,810, SD = $1,250 F = 1662.62 p < 0.0001). In addition, there were significant differences in osteoporosis medication costs when comparisons were made between members who were non-persistent and members who were persistent. These costs were significantly lower for non-persistent members (M = $2,391, SD = $1,746) than for persistent members (M = $10,516, SD $1,520 t =28.06 p < 0.0001).

### Association between teriparatide adherence and teriparatide persistence with fracture rates

**Figure 2:**
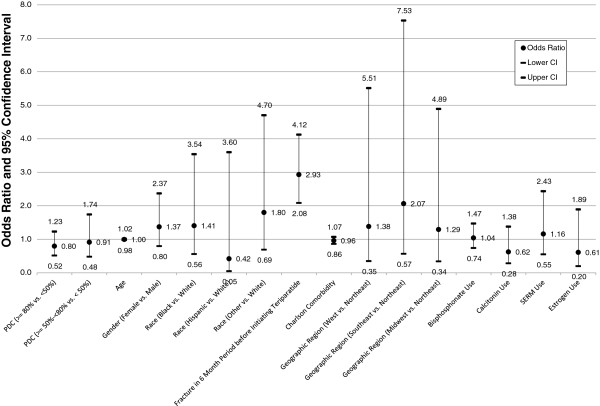
Association of Teriparatide Adherence and 12-Month Fracture Risk*. Likelihood Ratio: (Χ2 = 55.16, 16 d.f. p < 0.0001), c statistic: 0.669. *Odds ratio and 95% confidence interval of experiencing a fracture during 12-month follow-up period based on adherence and adjusted for age, gender, race/ethnicity, pre-period fractures, comorbidity, geographic region, and pre-period OP medication use.

**Figure 3:**
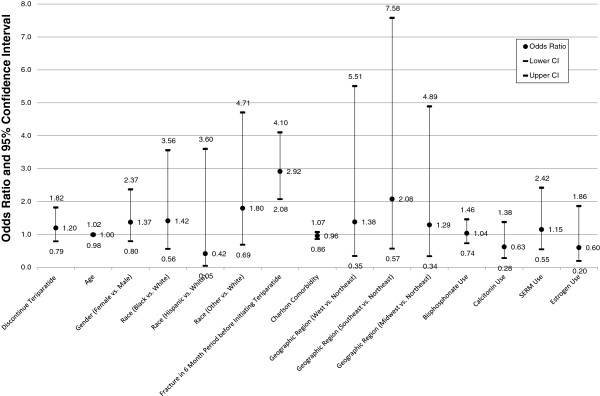
Association of Teriparatide Persistence and 12-Month Fracture Risk*^†^**.** Likelihood Ratio: (Χ2 = 54.83, 15 d.f. p < 0.0001), c statistic: 0.669. * Odds ratio and 95% confidence interval of experiencing a fracture during 12-month follow-up period based on persistence and adjusted for age, gender, race/ethnicity, pre-period fractures, comorbidity, geographic region, and pre-period OP medication use. ^**†**^Discontinue teriparatide = non-persistent with teriparatide.

Multivariate logistic regression analyses were used to assess the associations between adherence and persistence and the risk for a fracture in the 12-month period following teriparatide initiation. As noted in Figures 2 and 3, two separate models were evaluated. The results in Figure 2 describe the association of adherence and fracture outcome, and those in Figure 3 depict the association of persistence and fracture outcome. Age, gender, geographic region, race/ethnicity, prior osteoporosis medication use, Deyo-Charlson comorbidity, and prior fracture were covariates in both models.

As noted in Figures 2 and 3 and in Table 7, there were no significant differences in fracture outcomes whether or not patients were persistent in taking teriparatide (OR 1.2, 95% CI 0.79, 1.82, NS), or regardless of their adherence status (high versus low adherence [OR .81, 95% CI 0.515- 1.231, NS], and moderate versus low adherence [OR .91.95% CI 0.479, 1.741, NS]). While the regression models demonstrated no significant association between teriparatide adherence or persistence and 12-month fracture outcomes, the results indicated that patients who had fractures in the 6 months before initiation of TPTD were almost 3 times more likely to have a fracture in the follow-up period (OR 2.9, 95% CI 2.1-4.1 p < 0.0001). Furthermore, only 28% of members continued teriparatide use after their first fracture episode.

**Table 7:** Logistic regression of adherence and fracture risk* ^**†**^

**Variable**	**Odds ratio**	**95% confidence interval**	**P value**
PDC (≥ 80% vs. <50%)	0.81	(0.515, 1.231)	0.48
PDC (≥ 50% ≤80% vs. < 50%)	0.91	(0.479, 1.741)	0.94
Age	1.00	(0.976, 1.016)	0.66
Gender (Female vs. Male)	1.37	(0.796, 2.368)	0.25
Race (Black vs. White)	1.41	(0.558, 3.538)	0.48
Race (Hispanic vs. White)	0.42	(0.048, 3.599)	0.29
Race (Other vs. White)	1.80	(0.689, 4.703)	0.22
Fracture in 6 Month Period before Initiating Teriparatide	2.93	(2.084, 4.122)	<.0001
Charlson Comorbidity	0.96	(0.861, 1.07)	0.46
Geographic Region (West vs. Northeast)	1.38	(0.347, 5.513)	0.99
Geographic Region (Southeast vs. Northeast)	2.07	(0.566, 7.531)	0.05
Geographic Region (Midwest vs. Northeast)	1.29	(0.341, 4.889)	0.76
Bisphosphonate Use	1.04	(0.738, 1.469)	0.82
Calcitonin Use	0.62	(0.283, 1.378)	0.24
Selective Estrogen Receptor Modulator Use	1.16	(0.551, 2.433)	0.70
Estrogen Use	0.61	(0.197, 1.891)	0.39

* Results of multivariate logistic regression analyses, with ‘Fracture in 12 Month Post-Period’ as the outcome variable.

^†^ Among patients with a fracture.

## Discussion

This study explored the teriparatide utilization patterns of patients with Medicare Advantage with Prescription Drug (MAPD) coverage, excluding LIS beneficiaries, who initiated teriparatide therapy, and the manner in which those utilization patterns were associated with fracture outcomes and healthcare utilization. While the results suggested that there were no significant differences in fracture outcomes whether or not patients persisted with taking the drug or whether or they were adherent or not, this study identified specific patterns in utilization and cost in this population.

The results of the adherence component of this study support findings of other studies that have evaluated osteoporosis therapies. Similar to previous studies, these findings suggest that patients with osteoporosis tend to have poor adherence to or persistence with prescribed therapies, and that this poor adherence/persistence may be associated with adverse clinical and economic outcomes [24-27]. The results indicated that only 21% of the study group was optimally adherent (PDC ≥ 80%) to their teriparatide therapy at one year of follow up, and that only 24% of patients continued to take the drug during that same time period. Some studies have reported higher rates of adherence with teriparatide therapy 12 to 18 months following therapy initiation as compared to this study’s findings [28-30]. Arden and colleagues [29] reported an 87% persistence rate among 435 patients residing in the U.K., and Adachi et al [30] reported an 82% adherence rate in a cohort of 116 patients participating in an 18-month, multi-center prospective study of teriparatide adherence. In addition, a retrospective study by Yu et al [6] demonstrated an adherence rate of 81% in a cohort generated by the combined administrative claims of 3,587 commercial and Medicare patients in the U.S. The difference in these results may be due to the fact that the patient pool for this study was comprised solely of Non-LIS Medicare Part D beneficiaries, a large percentage of which had reached the coverage gap in the 12 months following initiation of teriparatide. The lack of funds to cover the cost of the medication once patients reach the coverage gap could explain the observed lower rates of adherence.

A surprising finding in this study was that less than half of the patient cohort (46.4%) had used another osteoporosis medication in the 6 months prior to initiating teriparatide therapy. In lieu of choosing to take an oral medication once weekly or monthly, a large percentage of patients initiated a once-daily injectable dosage form (teriparatide). Teriparatide is recommended for patients at high risk of fracture, and so initiation in treatment naïve patients does occur; however, common clinical practice is for teriparatide to be utilized after treatment failure on first line therapy. An additional surprising finding is the low incidence of prevalent fracture (50%). With teriparatide’s indication for use, one would have expected a much higher incidence of patients with prevalent fracture. In clinical practice, teriparatide may often be utilized after treatment failure on another osteoporosis medication [8,31]. This study’s analysis did not allow for the assessment of the level of severity of osteoporosis in the patients, which presumably would be more severe in this cohort based on the degree of initiation of teriparatide. It is possible that some health care providers may be prescribing teriparatide as a first line agent based on a comprehensive assessment of patients’ disease severity.

Interestingly, there was a low rate of persistence with teriparatide after the first observed fracture. In fact, only 28% of patients in the study continued using the drug after the first fracture; however, the underlying cause for this non-persistence could not be investigated due to the claims based structure of this study. It was not clear whether the non-persistence was due to patient apathy toward the benefit of their therapy, to patients attributing the causes of their fractures to treatment failure, or due to prescribers deciding to discontinue the therapy and opting for another treatment strategy.

The analyses evaluating the relationship between utilization patterns (adherence or persistence) and fracture-related utilization and costs revealed interesting trends. The results suggested that patients who were highly adherent or persistent with teriparatide therapy had lower rates of emergency room utilization. among patients who had poor adherence or non-persistence with teriparatide therapy. Conversely, the patient groups that were highly-adherent and/or were persistent with teriparatide therapy had higher overall and pharmacy costs than did their non-adherent/non-persistent counterparts; almost 70% of the total costs of these highly-adherent and persistent patients were attributable to pharmacy expenses.

Our finding of a higher risk of fractures among patients who had experienced fractures in the 6 months prior to teriparatide initiation is consistent with previous studies of patients with osteoporosis [32-34]. Klotzbuecher and colleagues’ review determined that patients with histories of previous fractures at different sites have a two-fold increased risk of having a future fracture [32]. Similarly, Haentjens and colleagues’ meta-analysis suggested that prior wrist or spine fracture was associated with an increased risk of future hip fracture in both men and women [33]. Furthermore, Lindsay and colleagues’ analysis of data from international trials of osteoporosis treatment estimated that among older women who had a vertebral fracture, 20% would experience another vertebral fracture and 26% would incur a vertebral or non-vertebral fracture within 12 months [34,35]. Our observations contribute to this pool of information and support the need to closely monitor and treat patients who have fractures in order to mitigate the risk of future fracture, which is known to increase morbidity and mortality rates [1,35,36]. Further research is warranted in order to better understand and reduce future risk, and to assess the efficacy of interventions for reducing the incidence of fracture among high-risk patients.

The present study has limitations that warrant consideration when interpreting the findings. This is an observational, retrospective claims-based study whose results are indicative of associations of medication-use variables with clinical and economic outcome variables; the results do not show causal relationships among variables. In addition, other limitations common to studies using administrative claims data apply to this study. These include threats to validity presented by missing data, errors in claims coding, a lack of data on indirect costs, as well as unmeasured factors which may be associated with adherence. For example, the study did not control for potentially confounding factors such as patient health beliefs and provider-patient communication, which could have obscured the relationships of adherence and persistence with the clinical and economic outcomes. Additionally, the observations around prevalent fractures as well as use among treatment naïve patients makes it difficult to extrapolate these data to one better defined by the approved indication for use. Although multivariate regression modeling is used in the present study to reduce selection bias and control for confounding, it can only reduce bias caused by measured covariates; it cannot reduce bias caused by unmeasured covariates. Other study limitations may be the results of the criteria used to define the sample as well as those used to evaluate fracture outcomes. Patients who were LIS beneficiaries during the study period (560 patients) were excluded from the sample. Had those patients been included, the sample size would have been much larger, potentially increasing the generalizability of the study findings and permitting comparisons of the characteristics of the present study’s patients with those of the LIS group. With regard to the assessment of fractures, the present study’s analysis excluded fractures occurring within the first 90 days of teriparatide initiation, similar to approaches used in previous studies (e.g. 180 days used by Halpern et al. [27] so as to allow sufficient time for treatment effects to begin [37]) and assessed adherence and fracture outcomes concurrently. Some researchers posit that these approaches may give rise to inaccuracies in determining the association of adherence with fracture outcomes, and suggest that it is advisable to include an assessment of adherence prior to the occurrence of a fracture [38,39]. Finally, the current study uses data from a single large national health insurance company exclusively, thus the results might not be generalizable to the general U.S. population.

## Conclusions

In summary, the study’s findings suggest a need to better understand and improve adherence to teriparatide in Medicare Part D beneficiaries with osteoporosis. The results were inconclusive in terms of the exact relationship between poor adherence and negative outcomes such as increased utilization of ER visits and fracture rates. The results demonstrated a trend for highly-adherent patients to have higher healthcare costs most likely due to significantly higher pharmacy expenditures. Furthermore, patients who persisted with teriparatide use had significantly higher healthcare costs than patients who were non-persistent with therapy. The results also suggested that emergency room utilization was higher among non-persistent patients and patients with low adherence; but that effect cannot be definitively tied to teriparatide use. In addition, the study’s results highlight the importance of monitoring clinical outcomes of patients who have fractures in order to reduce the risk of future fractures. Future studies could evaluate the underlying factors contributing to higher healthcare utilization rates as well as the factors underlying more favorable outcomes such as lower utilization rates (for example, regression analyses with ER visits and inpatient hospitalizations as outcome variables).

## Methods

### Study design

This retrospective cohort study was designed to analyze the medical and pharmaceutical claims data of 761 Medicare members who had both pharmacy and medical benefits. The study’s goals were to describe the clinical and economic outcomes of Medicare Part D beneficiaries newly started on teriparatide, and to examine the relationships of adherence and persistence with healthcare resource use and fracture outcomes. The data sources for this study included member enrollment, medical, and pharmacy data generated from the claims database of a large national healthcare provider. This study was part of a larger protocol, which was approved by the Western Institutional Review Board.

### Selection of participants

The patient sample was drawn from a cohort of patients from a large national health insurance company who were enrolled in the Medicare Advantage with Prescription Drug coverage (MAPD) plan. In addition, patients included in the study were Medicare Part D recipients, aged 18 to 89 years old, and not members of the Low Income Subsidy program (LIS) for Medicare Part D at any time during the study period (LIS is a Medicare program which provides additional prescription drug coverage to low income Medicare Part D beneficiaries). LIS members were excluded from the study because they were more likely to be buffered from the cost-sharing effects of the Medicare Part D plan. Study participants had at least one pharmacy claim for teriparatide (Generic Product Identifier [GPI] code 30044070) during the identification period of January 1, 2008 to December 31, 2009. The date of the first pharmacy claim for teriparatide was considered the index date. In addition, participants had at least 18 months of total enrollment, that is, at least 6 months prior to the index date (baseline period) and at least 12 months post the index date (follow-up period). The patients’ baseline demographics, pre-existing fracture rates, medication use, and comorbidities in the baseline period were evaluated. Clinical and economic outcomes were assessed during the 1-12 months post-index and the 1-24 months post–index.

### Primary outcome measures

#### Healthcare resource utilization

The quantity of both inpatient and outpatient services received, as well as all-cause health care resource utilization in the claims data, were measured during the 12 and 24 month follow-up periods. This included outpatient (physician office visits, procedures and tests), inpatient hospitalizations, emergency room (ER) visits, and pharmaceutical claims.

#### Healthcare costs

Total overall costs and total fracture-related health care costs (reimbursements and co-pays) associated with the aforementioned health care resource utilizations, were measured during the follow-up period. All pharmacy and medical claims for members were examined during the follow-up period to assess total out of pocket pharmacy and medical spending. Costs were adjusted to 2010 dollars using the U.S. Bureau of Labor Statistics’ Consumer Price Index for Medical Care. Fracture-related services were characterized by an ICD-9 code according to the criteria used to identify fractures, which are outlined in the fracture section below.

#### Fractures occurring in the 12 and 24-month follow-up period

Fractures occurring in the follow-up period were assessed, with the exception of fractures occurring within the first 90 days following teriparatide initiation, as those fractures could not be reliably associated with osteoporosis treatment. The following criteria were used to identify the presence of fractures:

##### Fracture inclusion criteria

Fractures were included if they were identified by the ICD-9-CM codes for fractures likely related to osteoporosis 813 to 814 (radius, ulna, and carpal fractures), 805 (vertebral fracture without spinal cord injury), 807.0 to 807.4 (rib fractures), 820 to 821 (femur fracture), or 808 to 809 (pelvic fracture and ill-defined fracture of the trunk). Also included were first fractures at any site identified by primary or secondary diagnosis (ICD 9) indicating a fracture on a non-diagnostic claim of inpatient, emergency room, or outpatient service, and ICD 9 codes (733.1) for pathologic fracture not related to cancer or Paget’s disease.

##### Fracture exclusion criteria

Fractures were excluded if they were identified by ICD 9 diagnosis codes associated with a malignant neoplasm (excluding non-melanoma skin cancer) or a benign tumor of bone in the 12 months before or in the 6 months following the date of the fracture, and if there was evidence for a malignancy that had diagnosis codes only for a history of cancer (ICD-9 V10). Pathological fractures were excluded if the patients were verified as having cancer within 6 months of the fracture diagnosis. Evidence of traumatic fracture (for example, fractures that were the result of a motor vehicular or pedal cycle accident), fractures accompanied by codes suggesting 3 or more simultaneous fractures, and open fractures, were excluded because they were likely to be the result of trauma rather than osteoporosis.

### Independent variables and covariates

#### Covariates

##### Age, gender and race/ethnicity

Age, gender, and race/ethnicity were captured from enrollment data. Patient age was measured as of the index date.

##### Geographic region

Geographic region was based on the patient’s state of residence on the index date. Regional assignment (Northeast, Midwest, South, and West) was based on the U.S. Census Bureau assignment of states to a geographic region.

##### Osteoporosis medication utilization

This was evaluated using the pharmacy claims for each member during the baseline and follow-up periods. Utilization was based on receiving one or more paid pharmacy claims where the MediSpan© Generic Product Identifier (GPI) code was for a bisphosphonate (GPI-6 30.04.20), calcitonin (GPI-6 30.40.30), selective estrogen modulator (SERM) (GPI-8 30.05.30.60), or estrogen replacement therapy (GPI-4 24.99, 55.35).

##### The Deyo-Charlson comorbidity index (DCI)

This index was used to assess the cohort’s baseline comorbid conditions. It uses 17 categories of comorbidity to calculate a score that reflects cumulative increased likelihood of one-year mortality. It is based on ICD-9 diagnoses and procedure codes, and their associated weights. The DCI score can range from 0 to 33. Claims with the specified codes are used in the calculation of the DCI if they meet the following criteria (1) Used on an inpatient hospitalization or (2) Two or more claims 30 days apart on separate outpatient claims [40].

##### Prior fracture

Fractures episodes occurring in the ‘pre’ period (6 months before initiation of teriparatide) were also assessed using the aforementioned fracture inclusion/exclusion criteria for identification of fractures, described above.

### Independent variables

#### Medication adherence – proportion of days covered (PDC)

Medication adherence was calculated as the proportion of days covered (PDC). PDC was measured as a 12 month fixed follow-up period from the index date. The PDC was calculated as a percentage, which is equal to the number of days with teriparatide on hand divided by the number of days in the period. A PDC of 100 will equal 100% adherence. The number of days with teriparatide on hand was calculated using a set of rules to avoid double-counting covered days when prescription fills for teriparatide overlap, as opposed to summing the day supply for all teriparatide prescriptions received during the period. Summing the days’ supply is used in the calculation of a Medication Possession Ratio (MPR), another adherence measure. This method can overestimate the level of adherence during a fixed period of follow-up (i.e. 12 months, as in this study), especially if early refilling of prescriptions by the patient frequently occurs. ‘Appropriate’ or optimal medication usage was defined as having a PDC ≥ 80% and ‘inappropriate’ or suboptimal medication usage was defined as having a PDC of <80%. For the purpose of comparison, PDC was divided into the 3 categories of high, moderate and low. ‘High’ was defined as a PDC greater than or equal to 80% (PDC ≥80%), ‘moderate’ was described as a PDC greater than or equal to 50% but less than 80% (50% ≤ PDC < 80%), and ‘low’ was a PDC less than 50% (PDC < 50%).

#### Therapy non-persistence

Non-persistence with therapy (discontinuation) was defined as at least a 60-day gap in any subsequent teriparatide medication claim. The discontinuation date was defined as the date of the last prescription claim prior to the refill gap being exceeded, plus the day of supply of that claim. The refill gap was calculated using a procedure to avoid double-counting covered days when prescription fills for teriparatide overlap [41].

### Other variables

#### Pain medication utilization

This was evaluated using the pharmacy claims for each member during the follow-up periods. Utilization was based on receiving one or more paid pharmacy claims where the GPI was for an opioid or non-steroidal anti-inflammatory drug (NSAID) therapy (GPI code 661000xx).

### Analysis plan

A sample attrition table was produced to identify how many members were removed from the sample due to the specified exclusion/inclusion criteria. Descriptive statistics (mean, standard deviation and range for continuous variables, as well as percentages for dichotomous variables) were produced to summarize the group’s demographic and clinical characteristics. Counts were provided (mean, median, standard deviation, and range) to summarize the overall and osteoporosis-related resource utilization and cost patterns for members with at least 12 months of post-period follow-up (i.e., months 1-12 post index). The same measures were calculated for members with 24-months of post-period follow-up (i.e., months 1-24, post index). Similar counts were produced and then grouped according to patients’ PDC status for comparison of 12 and 24 month outcomes. PDC calculations were used as predictor variables in analyses of 12 and 24 month clinical and economic outcomes. We summarized the 12 and 24 month overall and osteoporosis-related resource utilization and cost patterns between the groups of members based on their utilization of teriparatide. Chi Square analyses and ANOVA pairwise comparisons were performed to determine whether there were significant differences in healthcare utilization and costs for patients with high adherence (PDC ≥80%) as compared to those with low adherence (PDC < 50%). T-Tests were used to assess significant differences in utilization and costs for patients who were persistent with therapy as compared to those who were non-persistent.

Multivariate logistic regression analyses were used to assess the association between adherence/persistence and fracture outcomes. Two regression models were evaluated to separately assess the associations of a) adherence and b) persistence, on fracture occurrence at follow-up. In the first model, adherence status was divided into the categories of high versus low (PDC < 80% vs. PDC < 50%), or moderate versus low (50% ≤ PDC < 80% vs. PDC < 50%). For the second model, persistence with teriparatide was measured as dichotomous variable (non-persistence with treatment or persistence). In both models the dependent variable was the occurrence of a fracture during the 12-month follow-up period. Age, gender, geographic region, race/ethnicity, prior osteoporosis medication use, Deyo-Charlson comorbidity, and prior fracture were included as covariates in the models. All analyses were performed using SAS Enterprise Guide 4.2 statistical software (Cary, North Carolina, USA). The significance level was set at < 0.05 for all statistical analyses.

## Competing interests

LHF, AML and CLU are employees of Competitive Health Analytics, Humana Inc., which received funding from Eli Lilly and Company to conduct this study. SAF and RTB are employees and stockholders of Eli Lilly and Company which provided funding for this study and is the manufacturer of teriparatide.

## Authors’ contributions

LHF drafted the manuscript. AML collected the data and performed the statistical analyses. SAF and RTB came up with the concept of the study. LHF, AML, CLU, SAF and RTB participated in the design of the study, interpretation of the results, and in the progress and critical revisions of the manuscript. All authors read and approved the final draft.

## Pre-publication history

The pre-publication history for this paper can be accessed here:

http://www.biomedcentral.com/1471-2474/14/4/prepub
